# Addition of Care for Transgender-Related Patient Care into Doctorate of Pharmacy Curriculum: Implementation and Preliminary Evaluation

**DOI:** 10.3390/pharmacy6040107

**Published:** 2018-09-29

**Authors:** Cheyenne Newsome, Li-Wei Chen, Jessica Conklin

**Affiliations:** 1 College of Pharmacy and Pharmaceutical Sciences, Washington State University, PO Box 1495, Spokane, WA 99210-1495, USA; Li-wei.chen@wsu.edu; 2 College of Pharmacy, University of New Mexico, MSC 09 5360 Albuquerque, NM 87131, USA; jeconklin@salud.unm.edu

**Keywords:** pharmacy education, transgender, lesbian gay bisexual transgender (LGBT), active learning, flipped classroom, transgender education, health provider education

## Abstract

The number of transgender and gender-diverse patients seeking medical care in the United States is increasing. For many of these patients, pharmacotherapy is a part of their gender-affirming transition. Effective instructional methods and resources for teaching pharmacy students about this patient population’s social considerations and medical treatments is lacking. Three hours of educational material on caring for transgender patients was added to a third-year course in a four-year Doctorate of Pharmacy program in the United States. The content included cultural, empathy, and medical considerations. Students in the course were given a survey to assess their perception of each instructional method’s effectiveness and self-assess their confidence in providing competent gender-affirming care to transgender people before and after the learning session. The survey response rate was 36% (54/152). Students’ self-assessed confidence to provide competent care to people who are transgender increased significantly. Before the learning session, the median confidence level was 4/10 (Interquartile range (IQR) 3–6), after the class session, the median confidence increased to 7/10 (IQR 6–8, *p* < 0.01). Students rated the pre-class video, jeopardy game, and patient panel as most helpful to improving their skills. The addition of transgender-related patient care material into the Doctorate of Pharmacy curriculum significantly increased the students’ confidence to provide competent care to people who are transgender.

## 1. Introduction

A recent estimate indicates that the number of people in the United States who identify as transgender is 0.39%. Prevalence estimates of this population nearly doubled from 2009 to 2015 [1]. The increase in the number of individuals reporting they are transgender in these surveys may not be an actual increase in people who are transgender, but instead reflective of the increased social acceptance of this community that ultimately leads to more individuals who are transgender disclosing their gender identity [1]. Many people who are transgender seek hormone therapy as a part of their gender transition but do not obtain hormone therapy from medical providers because of various factors including history or fear of mistreatment by healthcare providers [2,3]. The increasing number of patients disclosing their gender identity and seeking care highlights the need for pharmacists to understand transgender patients and their healthcare needs [4]. Studies have found that between 23% and 71% of male-to-female (MTF) individuals reported recent use of hormones from a nonmedical source [2,5,6]. These findings are concerning because there are health risks associated with obtaining medications from nonmedical sources. These risks include using medications that are adulterated or misbranded, improperly using medications, and a lack of drug monitoring [7,8,9]. A survey of community pharmacists found that over 80% of the participants felt that community pharmacists play an important role in providing care for transgender patients, but less than 30% felt confident in their abilities to manage health concerns of transgender patients [10]. Pharmacists need to be educated about transgender-related care, to ensure patients have access to providers with knowledge and skills to supply appropriate medications for gender transition [11]. 

Researchers in healthcare education have recognized the value of addressing curriculum gaps related to Lesbian, Gay, Bisexual, and Transgender (LGBT) populations by proposing methods of including relevant content in medicine [4,12], nursing [13], physical therapy [14], and pharmacy [15]. While educators in healthcare have begun to increase their focus on LGBT individuals in the curriculum, pharmacy curriculum content related to this group was limited, and material specific to the transgender population was even more scarce [10]. Moreover, most of the LGBT materials that have historically been provided focused on cultural awareness and empathy, rather than pharmacotherapy and medical treatments. In addition to providing suggestions for content, previous work has shown the importance of incorporating information related to a pharmacist’s role in increasing access to care for this population [16,17]. The Healthy People 2020 initiative from The Department of Health and Human Services aims to improve the “health, safety, and well-being of LGBT individuals” [18]. The Healthy People 2020 guidelines also note the need for healthcare professionals to provide specific attention to LGBT patients to address current health disparities [18]. In November 2014, the Association of American Medical Colleges published guideline recommendations for implementation of curricular and institutional climate changes to improve health care for individuals who are LGBT or Gender Nonconforming. This guideline aims to provide medical schools with education about the health needs and how to integrate this content into the curriculum [19]. While the Accreditation Council for Pharmacy Education (ACPE) has not explicitly provided guidance for implementing transgender patient care into the curriculum, the 2016 ACPE Standards for Doctor of Pharmacy Programs include requirements for curriculums to include content regarding cultural awareness, healthcare systems, and pharmacotherapy [20]. This includes minority populations such as individuals who are transgender or gender diverse. There is little guidance and even fewer clinically trained clinical pharmacist who provide transgender-related education. A recent article by Ostroff et al. described the successful implementation of transgender-related education into a pharmacy therapeutics course using a mixed technique of lecture and patient videos. Ostroff et al. utilized a transgender-affirming provider for guidance in the curricular development, which not all educators will be able to access [21].

This research describes the design and content of course materials on transgender patient care, both social considerations and medical treatments. In addition, this article outlines publicly available resources that can aid educators in implementing transgender-affirming content into colleges of pharmacy’s curricula. This study investigates students’ perceived usefulness of the individual components of the curriculum to identify instructional methods that may be useful and the effect of this instruction on changing students’ confidence to provide care to individuals who are transgender. 

## 2. Materials and Methods

The topic of caring for transgender and gender-diverse patients was incorporated into a third-year Doctorate of Pharmacy course, i.e., Therapeutics of Special Populations. This course covered other topics, including considerations in geriatrics, ostomy, bariatric, pediatric, obstetrics, and hospice. An active learning approach was utilized for the entirety of the class, using a flipped classroom for course delivery as it is used throughout the entire PharmD program curriculum. The essential concept of flipped learning is student preparation before the learning session to develop foundational knowledge. There is minimal use of didactic lectures. Instead, the focus is on students completing preassigned readings or watching pre-recorded videos and then actively applying knowledge during class time [22,23,24]. Three hours were allocated to discuss medical and cultural considerations for people who are transgender or gender diverse in the Fall 2017 semester. The curriculum for this block was developed by a pharmacist faculty member who had previous clinical experience working with patients who are transgender. The learning objectives for the material are listed in Table 1. 

The pre-class material consisted of a 40-min video and a list of frequently asked questions and their answers. The pre-recorded video addressed topics such as terminology, health disparities, prevalence, and pronouns, along with pharmacotherapy treatments [3,25,26]. Areas of focus for the medications were dosing, formulations, desired and adverse effects of therapy, and contraindications for masculinizing and feminizing hormone therapies. The information on medical care was created from a review of guidelines for transgender and gender-diverse people [27,28]. The Frequently Asked Questions about Transgender People from the National Center for Transgender Equality was additional pre-class material for students to review [29]. 

The three hours of in-class time was divided into a two-hour active learning session and a one-hour panel discussion. The two-hour active learning session occurred first and consisted of a jeopardy game, viewing and discussing a video of a story about a patient who is transgender, student gender identity exploration exercise, and patient cases. The jeopardy game reviewed terminology and information from pre-class materials. This was an opportunity for students to gauge their knowledge and identify or clarify any concepts that were unclear. The video was narrated by a mother whose child is transgender and discussed challenges faced during the coming-out phase. The gender identity exploration activity was adapted from Lavender Health’s Gender Role Socialization activity. During this activity, students divided into groups and discussed their perceptions and experience of gender. They reflected on questions such as ‘When did you learn you were a boy or a girl?’ and ‘How did you know you were a boy or a girl?’ [30]. During the patient cases, students were split into groups of three and role-played being a patient and pharmacist, with the other student observing to provide feedback. The student playing the pharmacist was given a prescription for feminizing or masculinizing medications and instructed to counsel the patient. The student role-playing the patient was given some additional information and instructed to ask specific questions about the medications, if they were not addressed in the pharmacist’s counseling. When the session was complete, the students portraying the patient and observer gave feedback to the student who provided the counseling. This was repeated with an additional case, so other students could practice their counseling skills. 

A panel of transgender and gender-diverse individuals were included into the learning session, because studies indicate that direct interaction with patients helps health profession students form more positive impressions of marginalized patient populations [31]. Panelists were recruited through a local transgender community support group. The panel was composed of three voluntary participants: a transgender man, a transgender woman, and a gender non-binary person. The panel discussion began with the moderator asking panelists to introduce themselves. After these initial introductions, the open question-and-answer panel discussion began. Questions were posed by students in the class, and all students were respectful and asked appropriate questions. 

Students completed an electronic survey to evaluate the learning session. The survey had the student assess the impact of the course content and activities on students’ confidence in caring for transgender people and their perceived effectiveness of each element of instruction (Listing 1). The survey included four questions and was sent to the students via the online survey platform Qualtrics^TM^ at the conclusion of the transgender panel activity. The first three questions were Likert-scale responses, and the last question was a free text box where students could leave feedback on the learning session. A Mann–Whitney U Test was performed to calculate statistical significance for change in student confidence to provide care to patients who are transgender. Statistics were completed in Microsoft Excel version 16.16.2 (Redmon, WA, USA). The Washington State University Office of Research Assurances found that the project was exempt from the need for institutional review board evaluation. 

**Listing 1.** Questions Included in the Survey.

(1) Before class this week, how confident were you in your abilities to provide patient care to people who are transgender? (0–10, with 10 being highest) (2) After class this week, how confident were you in your abilities to provide patient care to people who are transgender? (0–10, with 10 being highest) (3) Rate how helpful the following activities were in improving your abilities to provide care for people who are transgender (not helpful, somewhat helpful, very helpful, not applicable) - Pre-Class recorded video - Jeopardy game - Patient Video from YouTube - Gender Exploration - Patient Counseling Cases - Transgender Panel Discussion (4) Please provide feedback on the transgender learning sessions (most effective aspects and areas for improvement)

## 3. Results

**Figure 1:**
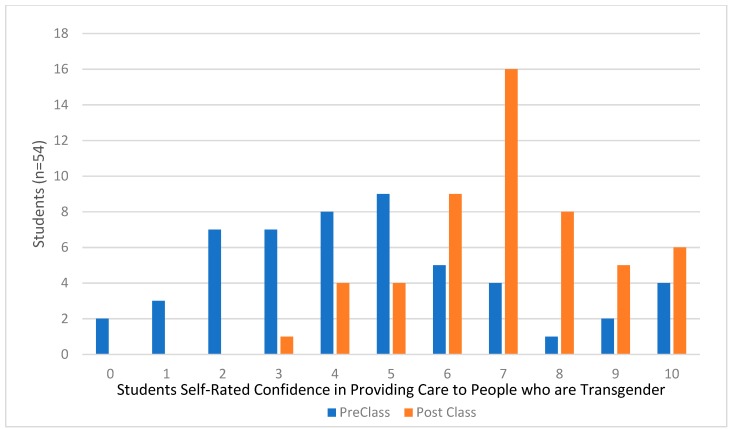
Confidence in Abilities to Provide Care to Transgender Patients (*n* = 54).

**Figure 2:**
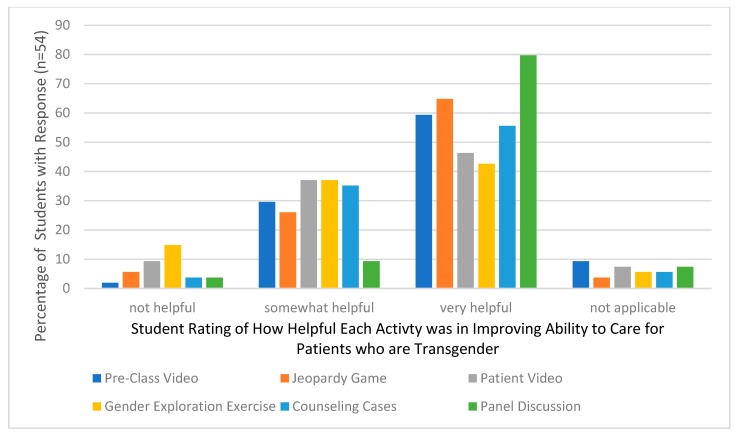
Student Perceptions on Effective Elements of Teaching (*n* = 54).

Fifty-four out of the 152 students enrolled in the course completed the survey. Students’ self-assessed confidence in providing care to patients who are transgender increased significantly. Before the learning session, the median confidence level was 4/10 (IQR 3–6), after the class session, the median confidence increased to 7/10 (IQR 6–8, *p* < 0.01). The distribution of responses is displayed in Figure 1. The responses for the perceived usefulness of the learning activities are displayed in Figure 2. Over half of the students who responded found the panel discussion, patient cases, pre-class video, and jeopardy game very helpful. Of all the activities, the gender exploration exercise was rated as the least helpful by the students.

Twelve students left free text comments, and 11 of them commented on the panel. Students’ free text responses are listed in Table 2. Three students wished the panel section would have been longer, and two stated they wished panel members would have discussed more about their medications. 

## 4. Discussion

The learning activities added to the course were effective at increasing student confidence in providing care for patients who are transgender. Many of the students rated the panel as very helpful. This emphasizes the impact of meeting individuals from different backgrounds can have on understanding and empathizing with people who present differently from what you are used to. Lack of understanding by healthcare providers is a common barrier to care for people who are transgender, which can lead to providers’ discomfort and fear. Learning about actual patients and having conversations are effective methods to help dissolve these barriers and fears [32]. A panel session can be highly effective and can be organized by faculty without any direct experience or extensive knowledge of transgender patient care. While in this discussion panel no students asked any inappropriate questions to the panel, it may be advisable that a faulty member screen questions or have students send in questions for the faculty member to read. This would allow for common themes to be addressed and also for students to have questions answered that they may feel uncomfortable asking in front of their peers. Volunteers can be recruited at local, state, or national transgender resource centers or support groups. If a panel is unable to be organized, videos from YouTube may be screened to determine an appropriate selection to give students insight into experiences of people who are transgender. 

While students’ confidence significantly increased, there were still some students whose confidence remained less than 5/10, indicating they were still not confident that they could provide quality care to someone who is transgender. Based on the feedback of this survey, there will be changes to the class materials to enhance the learning for future iterations of this learning session. The learning activity which fewest students found helpful to learning was the gender identity exploration exercise. This activity will be re-evaluated and may be shortened in duration. Also, the panel discussion will include a specific question about interactions with pharmacy and medication use. The patient cases will also be re-examined and peer-reviewed by others with experience in caring for people who are transgender.

This study had several limitations. First, the survey was given after the end of a learning session, and one question asked the students to self-assess their level of confidence prior to the learning session. This could have potentially led to recall bias. Second, the data was analyzed for the group as a whole instead of paired between students for the change in confidence level. The flipped classroom model worked well in this environment, but it may have been due to the students’ familiarity with this pedagogical style in the rest of their curriculum. In courses or curriculums that do not routinely employ flipped classroom teaching, instructors may experience difficulty getting students to spend time reviewing pre-class materials. These programs could still incorporate the active learning activities presented in this article as part of a didactic lecture session.

For institutions who are interested in incorporating transgender health education into their curriculum, there are many online resources. In this study, the Frequently Asked Questions about Transgender People from the National Center for Transgender Equality were provided to students for background prior to class. This handout is beneficial, as it explains in simple terms many questions people have but may not feel comfortable asking or may not know how to ask and provides a good foundation to expand upon. It can also provide a good foundation for educators interested in adding this topic into their program’s curriculum. It is available free online at: https://transequality.org/issues/resources/frequently-asked-questions-about-transgender-people. A great resource for developing the information about pharmacotherapy for gender transition is the World Professional Association for Transgender Health Standards of Care [28]. These guidelines have a chapter on hormone therapy discussing effect, risks, monitoring, and considerations for initiation. Additionally, TransEd is an online training program for healthcare students designed to provide improvements in student confidence in caring for people who are transgender. The program is freely available to interested healthcare programs and requires completion of a short Google form to gain access (http://www.transeducation.ca/). The program has modules that cover terminology, clinical best practices, and medications. There are recorded interviews with transmen, transwomen, and healthcare providers specialized in trans healthcare for learners to view. TransEd provides a facilitators’ guide to assist educators in identifying ways to incorporate the topic of care for people who are transgender or gender diverse into their existing curricula. Interested individuals may request access to the TransEd module by completion of a short Google form at http://www.transeducation.ca [33,34].

## 5. Conclusions

Addition of materials on transgender pharmacy care improved students’ perceptions of confidence in caring for transgender patients, with a panel discussion reported as the most helpful component of the learning session.

## Figures and Tables

**Table 1:** Learning Objectives.

Pre-Class Learning Objectives
(1) Define vocabulary used in caring for transgender and gender-diverse patients (2) Recognize health disparities for transgender and gender-diverse people (3) Identify when/how to ask someone what their pronoun is (4) Identify the mechanism, side effects, and contraindications to medications used in management of gender dysphoria (GD) (5) Identify benefits and risks of medications used for GD.
In-Class Learning Objectives
(1) Use appropriate vocabulary to care for transgender and gender-diverse patients (2) Respectfully ask patients about pronoun preference (3) Counsel patients on effects of medications used for gender dysphoria (4) Express empathy toward patients who face stigmas

**Table 2:** Free Text Responses to the Request, “Please provide feedback on the transgender learning sessions (most effective aspects and areas for improvement)”.

I didn’t learn anything medically related to their therapies; I feel that a lot of people were afraid to ask questions since the panelists focused on how people offend them
I wish the transgender panel was longer
n/a—I wish the panel was longer though. It was very interesting.
I think the patient panel was pretty useful. I would suggest the patients talk about their medications and specific side effects more though rather than almost completely about their life story. That was pretty interesting tho’ and I will be able to apply it to practice.
I would prefer the jeopardy game be more of a team activity than individual. Also during the panel, I felt one panelist made it not fine to “mess up” and I appreciated how the other two were more open to saying as long as you try or apologize then it will be fine.
The panel discussion was a really helpful tool to further understand the material. It was helpful to have real patients give their experiences and helpful advice on how to appropriately and effectively approach and discuss the topic without offending them.
The least effective activity this week was the Small Group Discussion on Gender because no one really knew what to say. We all just kind of sat there like, “I don’t know when I knew what I was I just was.”
the patient panel was extremely helpful and enlightening and gave me tips for how to make things less awkward
The panel was great. I appreciated the counselling case because some people felt a little awkward but it was an open environment to work out any issues.
I wish we had used the longer class period for the panel discussion and the shorter class period for small group discussion and counseling cases
I understand the panel discussion and the opinions they provided, but it was a very biased liberal based panel. It’s not your fault, but this topic is very opinion oriented and hard to capture.
Though the panel was very insightful, it would’ve been great to hear about the panel members’ experience with medications.
